# Weight-based discrimination in financial reward and punishment decision making: causal evidence using a novel experimental paradigm

**DOI:** 10.1038/s41366-022-01109-z

**Published:** 2022-03-25

**Authors:** Andrew Jones, Charlotte A. Hardman, Niamh Devlin, Charlotte R. Pennington, Eric Robinson

**Affiliations:** 1 Psychology, University of Liverpool, Liverpool, UK; 2 School of Psychology, Aston University, Birmingham, UK; 3 Institute of Health & Neurodevelopment, Aston University, Birmingham, England

**Keywords:** Health care, Public health

## Abstract

**Background/Objectives:**

Cross-sectional research has demonstrated weight-related stigma and discrimination, however experimental research providing causal evidence of financial-based weight discrimination is lacking. The aim of these preregistered experiments was to examine whether a novel paradigm in which participants attributed financial rewards and punishments could be used to detect weight bias.

**Subjects/Methods:**

One-hundred and twenty-one individuals participated in experiment 1 and one-hundred and sixty-six individuals participated in experiment 2. Both studies were conducted online, and participants were provided with biographies of hypothetical individuals in which weight-status was manipulated (normal weight vs. overweight/obesity) before being asked to provide rewards and punishments on their cognitive performance. In experiment 1 (within-participants design) participants observed one individual they believed to be normal weight and one individual they believed to be overweight/have obesity. In experiment 2 (between-participants design) participants observed one individual whilst also being provided with information about food addiction (Food addiction is real + individual with overweight/obesity vs. food addiction is a myth + individual with overweight/obesity vs control + individual with normal weight).

**Results:**

In experiment 1, participants punished individuals who were described as having overweight/obesity to a greater extent to individuals who were normal weight (Hedge’s *g* = −0.21 [95% CI: −0.02 to −0.41], *p* = 0.026), but there was no effect on rewards. They were also less likely to recommend individuals with overweight/obesity to pass the tasks (X^2^(1) = 10.05, *p* = 0.002). In experiment 2, participants rewarded individuals whom they believed were overweight/obese to a lesser extent than normal-weight individuals *(g* = 0.49 [95% CI: 0.16 to 0.83]. There was no effect on punishment, nor any impact of information regarding food addiction as real vs a myth.

**Conclusion:**

Using a novel discrimination task, these two experiments demonstrate causal evidence of weight-based discrimination in financial decision making.

## Introduction

Despite overweight and obesity having increased in recent times [1], many individuals with overweight or obesity experience public stigma, defined as ‘ the social rejection and devaluation that accrues to those who do not comply with prevailing social norms of adequate body weight and shape [2]’. Observational research has demonstrated that the experience of weight-related stigma has increased in line with the prevalence of overweight/obesity [3], and body weight is now stigmatised to a similar extent as race and gender [4]. Alongside observational research examining self-reported experiences of weight discrimination, research has also shown that individuals with obesity are blatantly dehumanised; Kersbergen and Robinson [5] demonstrated that individuals with obesity were considered less evolved and less human than individuals without obesity (*dz* ~ 0.51), and this effect was persistent across cultural contexts. Individuals who experience stigma may also internalise these negative attitudes [6], triggering problematic eating behaviours [7], and contributing to further weight gain (see the cyclic obesity/weight-based stigma model [8]).

Stigmatising beliefs may also lead to discrimination (also known as weight-bias), and the prevalence of weight-biases has also increased dramatically. Sikorski et al. [9] estimated the overall prevalence of reported weight-related discrimination in a representative European sample at ~7.8%, with similar estimates in English [10], and North American samples [4]. Commonly reported types of weight-related biases include disrespectful comments, poorer treatment in healthcare and education settings, poorer or denied service, and threats/harassment [11–13]. Furthermore, meta-analyses have demonstrated negative associations between higher body weight and workplace-related outcomes, such as lower likelihood of hiring [14] and lower performance evaluations [15], with some evidence that effects are moderated by gender (i.e. weight-biases disproportionately impact females; [16]. Taken together, these findings may contribute to the robust association between obese weight status and reduced income (SMD = –0.15 [–0.30, –0.01]; [17].

However, there is little experimental evidence examining the causality of weight bias. In entirely hypothetical scenarios, individuals with obesity were less likely to be accepted for university by members of the public [18]; less likely to be hired for a managerial role; experience helping behaviour [19]; or be selected for child adoption [20]. The potential effects of weight biases and discrimination are significant as individuals who report discrimination based on their weight are ~2.5x more likely to experience mental health disorders [2]. Experimental evidence in the general public is therefore largely hypothetical and focuses on decisions regarding denial of services (e.g. hiring, helping, adoption), as opposed to financial decision making. To our knowledge, there have been few attempts to directly examine discrimination in the general public who are led to believe their decisions impact the financial status of a real individual (non-hypothetical). Therefore, this study developed a novel experimental task to examine whether individuals would directly discriminate against individuals whom they believed to have overweight/obesity vs. normal weight when making financial decision. Specifically, we examined the role of weight discrimination in relation to financial rewards or punishments using a novel task across two experiments.

## Experiment 1

In Experiment 1, we hypothesised that participants would: (i) reward individuals whom they believed to have overweight/obesity less than normal-weight individuals and (ii) would punish individuals whom they believed to have overweight/obesity more than normal-weight individuals. The study design and analysis plan were pre-registered on the Open Science Framework in advance of data collection [https://osf.io/pqzgs]. (Note, our registration documents reported our analyses would be conducted in SPSS, however, we chose to conduct them in R as it is free and open-source allowing greater reproducibility.).

### Method

#### Participants

One-hundred and thirty-four participants took part in an online study; of those, 121 completed the study (95 females and 26 males; with 87.6% aged between 18 and 25). Participants were largely recruited from the student and staff population at the University of Liverpool, UK. Inclusion criteria was age 18+ years, and individuals with a current or previous diagnosis of a psychiatric disorder were excluded. The study was approved by the local research ethics committee (ref: 5516). Our a-priori power calculation estimated 139 participants (one-tailed: within subjects *t*-test) would be required to detect *dz* = 0.25 at 90% power. However, due to time constraints we were only able to recruit 121 participants, as this was a student-led project. Note, that if using more conventional statistical power (80%) we had sufficient statistical power to detect the desired effects with this sample size (*N* = 101).

### Materials

Novel financial discrimination task: Participants were shown a landing page that informed them that they would supervise the performance of individuals who were taking part in a course to improve their cognitive abilities (we named ‘Psy-Learn’), which was our cover story. They were specifically informed that the study was testing whether the administration of small financial rewards and punishments improved cognitive learning. The task then instructed them to observe individuals’ performance on several cognitive trials and allocate a small financial reward or punishment based on their performance on each trial. They were also told that they would also provide an overall judgment for the individual’s suitability for progression on the course.

Participants then provided consent and their basic demographic information. Following this, they were shown a sham screen which was designed to simulate Psy-Learn searching and connecting to an appropriate learner. Once connected, the participant saw the learner’s profile which included answers to the same demographic questions they had been asked for 60 s. They then observed the learner’s performance on six cognitive trials. These trials included a speeded reaction time (responding to an arrow which appeared on the screen in <500 ms), solving a 7-letter anagram, and a 7-word short-term memory test. Example trials and screen shots are shown in supplementary materials. Importantly, the participant was informed if the learner was correct or incorrect after each trial. If the learner was correct the participant was informed ‘The learner was CORRECT! How much will you REWARD them?’ and if the learner was incorrect, the participant was informed ‘The learner was INCORRECT! How much will you PUNISH them?’. They were then given a sliding scale ranging from 0 to 100 pence. The task was pseudo-randomised in that each learner got three trials correct and three trials incorrect, therefore our main dependent variables were the total reward and punishments allocated to the learner (0–300 pence). After providing a reward or punishment on the final task participants were given the option ‘Overall, would you recommend the participant be permitted to continue to the next stage of Psy-Learn in the future? They would be able to earn more money in future sessions. [YES, NO]’ and then asked if they would like to provide any feedback on their performance via an open-ended text box. They were then asked three memory questions based on the learner’s demographic information, with the key question ‘What was the learner’s weight? [Underweight, Average Weight, Overweight / Obese, Preferred not to say]. This served as a manipulation check to ensure the participant was paying attention to the information.

Following this, participants were then taken to back to the sham screen to connect to a second learner. Again, they saw the demographic information of the second learner for 60 s. All demographic information except weight status was kept constant between the first and second learners. Then participants observed and provided reward and punishment on the same six tasks, in which the second learner got three correct and three incorrect, however their performance was different than the first (e.g. different responses were made, and they got different questions correct / incorrect) to ensure the manipulation was not obvious. They were asked whether the participant should progress through the course and if they had any feedback on their performance. Finally, their memory of the second learner’s demographic information was assessed, critically this included memory of the learner’s weight. This then signalled the end of the study; participants were debriefed, and the cover story was explained. Across both studies, reward and punishment behaviour by the participants on individual trials demonstrated acceptable internal consistency (ω’s > 0.81: see supplementary online materials).

#### Procedure

Participants clicked a hyperlink to the study (hosted by Inquisit Web v.5, Millisecond, Seattle). They observed the cover story (information about Psy-Learn), and then provided informed consent. They were then asked to provide some categorical demographic information. They then saw the profile of a learner and completed the novel discrimination tasks, before remembering demographic information about the learner. After this, they observed and rewarded/punished the second learner, before being asked to recall the demographic information. The weight status of the learners (normal weight vs. overweight/obesity) was counterbalanced randomly. However, within participants the remaining demographic of the learners were closely matched (e.g. the age was within ±2 years, the gender was the same) to reduce any likelihood of discrimination on other demographic characteristics. To increase the credibility of the cover story, if participants clicked the link outside of the hours of 10 am to 10 pm, they received the message ‘None of our learners are online right now, please try again later’. The experiment lasted ~15 min.

#### Data reduction and analysis

Our main dependent variables were total reward and punishment [0–300 pence] separate for learners who were believed to be ‘normal/average’ weight and ‘overweight/obesity. We conducted paired samples *t*-tests separately for reward and punishment. We also examined these comparisons in participants who passed the manipulation check of recalling the learner’s weight status. One-hundred and eleven participants (91.7%) correctly remembered the normal-weight learners weight status and one-hundred and fourteen (94.2%) participants correctly remembered the overweight/obese learners weight status. Overall, 106 (87.6%) remembered both. There was one outlier in punishment scores (see online supplementary materials), but removal of this outlier or recoding the outlier to the closest non-outlying value (300 > 280) did not substantially influence the results (*p*s < 0.05). Therefore, analyses are presented with the outlier included. We conducted McNemar’s test to examine if the decision to progress was significantly different between the conditions. In supplementary materials, we examined the magnitude of reward or punishment in normal-weight participants only as an exploratory analysis. Data and analysis code is available on OSF, as is an example script for the experimental paradigm [https://osf.io/p2mtz/].

### Results

#### Participant demographics

A full breakdown of participant demographics is shown in Table 1. The sample largely comprised young, female students of higher educational levels and self-reported ‘average’ body weight.

**Table 1:** Legend: Demographic breakdown of participants in Experiment 1.

Demographics	*N*	(%)
Age		
18–25	106	87.4
26–35	6	5.0
36–45	6	5.0
46–55	3	2.5
Gender:		
Male	26	21.5
Female	95	78.5
Occupation:		
Full-time employed	9	7.4
Part-time employed	6	2.5
Student	105	86.8
Unemployed	1	>1
Education:		
GSCE/equivalent	4	3.3
A-level/equivalent	66	54.5
Degree or above	51	42.1
Self-reported Weight:		
Average weight	101	83.5
Overweight/Obese	18	14.9
Underweight	1	>1
Prefer not to say	1	>1
Alcohol use:		
Light drinker	80	66.1
Heavy drinker	31	25.6
Teetotaller	9	7.4
Prefer not to say	1	>1
Smoker:		
No	111	91.7
Yes	10	8.3

#### Rewards

There was no significant difference in reward between the normal weight (mean = 199.84, SD = 72.64) and overweight/obesity condition (mean = 205.34, SD = 73.58: *t*(120) = 1.25, *p* = 0.214, *g* = *−*0.07 [95% CI: *−*0.19 to 0.04]). When removing individuals who did not remember the learner’s weight status (normal weight: mean = 204.64, SD = 73.19; overweight/obesity: mean = 208.85, SD = 74.53), the effect remained non-significant (*t*(105) = 0.91, *p* = 0.367, *g* = *−*0.06 [95% CI: *−*0.18 to 0.07]).

#### Punishment

**Fig. 1:**
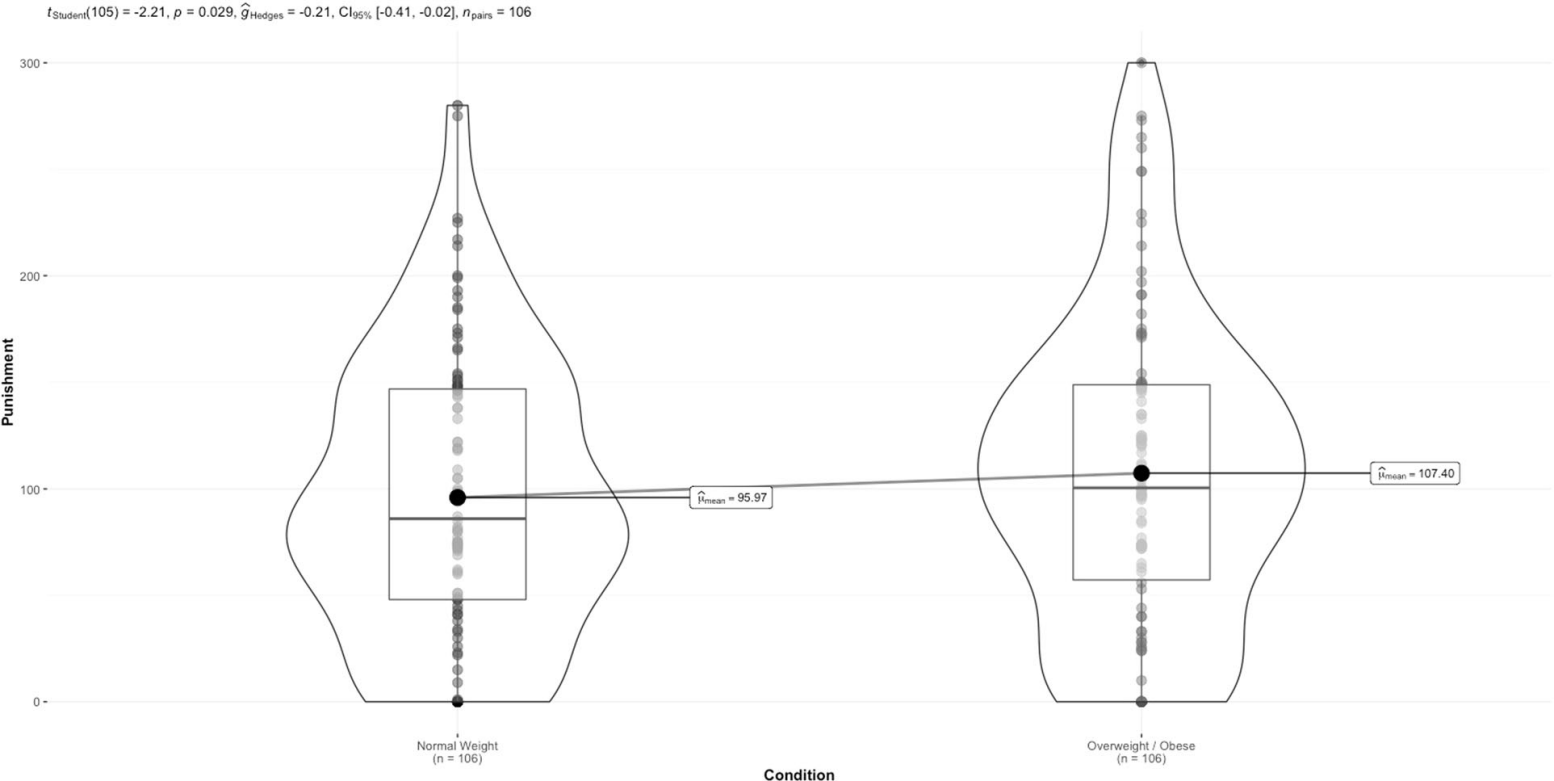
Differences in punishment across learner condition (normal vs overweight/obesity), in experiment 1. Points are individual data points. Boxplot represents the median and interquartile range. Violin plot represents the distribution of the data points.

There was a significant difference in punishment between the normal weight (mean = 96.79, SD = 66.31) and overweight/obesity conditions (mean = 107.50, SD = 71.06: *t*(120) = 2.28, *p* = 0.025, *g* = *−*0.15 [95% CI: *−*0.29 to *−*0.02]), whereby participants punished learners with overweight/obesity to a greater extent than normal-weight learners. When removing individuals who did not recall the learner weight status, the effect remained significant (*t*(105) = 2.21, *p* = 0.029, see Fig. 1).

#### Decisions to allow learners to progress

Participants recommended learners described as normal/average’weight to pass 75/106 (70.7%) of the time, and learners described as having overweight/obesity to pass 66/106 (60.2%) of the time. McNemar’s test was significant (X^2^(1) = 10.05, *p* = 0.002) demonstrating a significant difference in pass ratings: participants were more likely to recommend that normal-weight individuals pass onto the next stage than individuals with overweight/obesity, despite both getting the same number of tasks correct and incorrect.

#### Interim summary

In our novel experimental paradigm, participants provided greater punishment to individuals whom they believed had overweight/obesity compared to individuals who had normal weight and were less likely to recommend individuals with overweight/obesity to pass onto the next stage. This observation supports previous studies demonstrating that students willingly financially punish individuals [21], when the punishment has no direct benefit (known as altruistic punishment). Here, this might be exacerbated by increased willingness to punish an ‘outgroup’ [22], as most of our sample self-identified as average weight. A limitation of this study was the homogenous young, female, student sample, however. Given the majority student sample and within-participant design, there may have been some incidence of demand characteristics [23]. As such, we aimed to reduce this by using a between-subjects design in Experiment 2.

## Experiment 2

We attempted to replicate and extend the findings from Experiment 1 by also examining if weight-related discrimination was sensitive to information about food addiction as a causal explanation of obesity. Beliefs about the causality of obesity are thought to be highly influential in attribution of stigma and weight-related biases [24, 25]. Stigma is thought to be increased when individuals believe weight is a result of typical stereotypes of obesity (e.g. lack of willpower, inactivity and overeating [26, 27]). However, contradictory findings demonstrate that that attribution of a disease label may also reduce stigma and discrimination as it challenges the negative stereotypes above [28], see Latner et al. [29].

We hypothesised that (i) individuals who were believed to have overweight/obesity would be rewarded less and punished more than normal-weight individuals; and (ii) there would be a significant difference in reward and punishment between groups who were given information that food addiction was a real phenomenon versus those who believed food addiction was not a real phenomenon (non-directional). This study was preregistered on the OSF [https://osf.io/qjb65].

### Methods

#### Participants

One hundred and seventy-two participants attempted the study, with six failing to complete all parts. Participants were recruited from the staff and student population at the University of Liverpool (*N* = 87) and Prolific Academic (*N* = 79) to increase the diversity of the sample. Prolific is a crowdsourcing platform shown to provide high quality data [30]. Inclusion and exclusion criteria were the same as Experiment 1. Our a-priori power calculation indicated that 159 participants would be required to detect an effect size of *f* = 0.25 (*d* = 0.50) based on a between-participant ANOVA, with 80% power. We reasoned that the manipulation of food addiction beliefs would increase the effect size compared to Experiment 1.

### Materials

#### Experimental manipulations

Participants were randomly allocated to view one of three news articles, prior to the novel discrimination task. These articles provided information that (i) food addiction is real, (ii) food addiction is a myth, or (iii) climate change was a human-driven phenomenon (control message). The two articles on food addiction were taken directly from Hardman et al. [31] and are presented in the supplementary materials.

#### Novel discrimination task

The task remained the same as Experiment 1, however participants only observed one hypothetical learner. In this case, we adopted a between-participants design as it was unfeasible to provide participants with conflicting information (Food addiction is real vs Food addiction is a myth) to influence their beliefs in a within-participants design. Again, the learner pseudo-randomly got three questions correct and three incorrect.

#### Procedure

Participants clicked the link to the study, observed the cover story, and provided informed consent. They were then randomised to one of three experimental groups (Control message + normal-weight learner (*N* = 55); Food Addiction Myth message + overweight/obesity learner (*N* = 54); Food Addiction Real message + overweight/obesity learner (*N* = 57). Following this, they were provided with the text: ‘To ensure you are paying attention throughout the supervision and as a short measure of your own cognitive performance you will be shown the text from a recent newspaper story […] You will be asked questions about it at the end’. They were then shown the newspaper story for a minimum of 45 s, before being allowed to continue. Participants then completed the same demographic information as Experiment 1.

Following this, they were shown the sham loading screen before connecting to the learner and observing their cognitive performance on the six trials. In this experiment all learners were young females. Upon completion of this, they were asked to decide whether the participant was able to progress or not [YES, NO] and provide any feedback. Participants were then asked to recall the learner’s weight status, followed by a question on the newspaper article they observed (e.g. ‘In the news article you read the main theme was that ‘A growing body of scientists have found evidence that… [Climate change is caused by human activity, Food is not an active substance, Food can be as addictive as alcohol and drugs, Flying cars will be ever present in the next 10 years, India will be the next global superpower]. Finally, participants were debriefed. Each experimental session lasted ~10 min Local participants took part for course credits, and those who participated via Prolific were paid ~£1.00.

#### Data reduction and analysis

Our main dependent variables were total reward and punishment [0−300 pence] separate for the three experimental groups. We conducted a one-way ANOVA on both reward and punishment across the three experimental groups with Holm-Bonferroni corrections to follow up any significant main effects. To examine the effect of weight status we collapsed the Food addiction Myth and Real groups (both had a learner with overweight/obesity) and compared these to the control group (which had a normal-weight learner). We also examined these comparisons in participants who passed the manipulation check of recalling the learner’s weight status, but also the theme of the experimental message. Chi-squared tests were used to examine the association between experimental groups and the decision to allow course progression.

Memory for the learners’ weight status was high (*N* = 157: 94.6%). All participants correctly remembered the news article. There were no outliers for reward, but 5 outliers for punishment (>243 pence: see online supplementary materials for box plots). Removal or recoding of these outliers did not influence findings (all *p*s > 0.05) so we present the analyses with these outliers retained. Data and analysis scripts can be found here [https://osf.io/6pdfz/].

### Results

#### Participant demographics

A full breakdown of participant demographic information is shown in Table 2, split by sampling strategy (local vs Prolific).

**Table 2:** Legend: Demographic breakdown of participants in Experiment 2, split by sampling strategy.

	Local recruitment		Prolific recruitment		Total	
Demographics	*N*	(%)	*N*	(%)	*N*	(%)
Age						
18–25	62	71.3	26	32.9	88	53.0
16–35	15	17.2	19	24.1	34	20.5
36–45	3	3.4	14	17.7	17	10.2
46–55	4	4.6	14	17.7	18	10.8
56–65	3	2.4	6	7.6	9	5.4
Gender:						
Male	25	28.7	25	31.6	50	30.1
Female	62	71.2	54	68.4	116	69.9
Occupation						
Full-time employed	10	11.5	30	38.0	40	24.1
Part-time employed	10	11.5	13	16.5	23	13.9
Retired	2	2.3	3	3.8	5	3.0
Student	64	73.6	18	22.8	82	49.4
Unemployed	1	1.1	15	19.0	16	9.4
Education						
GSCE/equivalent	5	5.7	9	11.4	14	8.4
A-level/equivalent	42	48.3	15	19.0	57	34.3
MSc/equivalent	0	0	11	13.9	11	6.6
Degree or above	40	46.0	42	53.2	82	49.4
No formal qualifications	0	0	2	2.5	2	1.2
Self-reported Weight:						
Average weight	68	78.2	51	64.6	119	71.7
Overweight/Obese	14	16.1	24	30.4	38	22.9
Underweight	4	4.6	4	5.0	8	4.8
Prefer not to say	1	1.1	0	0	1	0.6
Alcohol use						
Light drinker	48	55.2	44	55.7	92	55.4
Heavy drinker	27	31.0	9	11.4	36	21.7
Teetotaller	12	13.8	24	30.4	36	21.7
Prefer not to say	0	0	2	2.5	2	1.2
Smoker						
No	73	83.9	68	86.1	141	84.9
Yes	13	14.9	11	13.9	24	14.5
Prefer not to say	1	1.1	0	0	1	0.6

#### Reward

A one-way ANOVA with three levels (control message + normal-weight learner vs. food addiction real message + overweight/obesity learner vs. food addiction myth message + overweight/obesity learner) was significant (F(2108.15) = 7.71, *p* < 0.001, *W*^*2*^*p* = 0.11 [95% CI: 0.02 to 0.22]. Pairwise comparisons indicated a significant difference between the control message + normal-weight learner group and the Food Addiction Myth message + overweight/obesity learner group (*p* = 0.001), whereby participants who received the food addiction was a myth message combined with an individual with overweight provided smaller rewards compared to the control message with an individual who was normal weight. There were no significant differences between the Control message + normal-weight learner group and Food Addiction Real message (*p* = 0.257), or the Food addiction real message and the food addiction myth message ((*p* = 0.129).

**Fig. 2:**
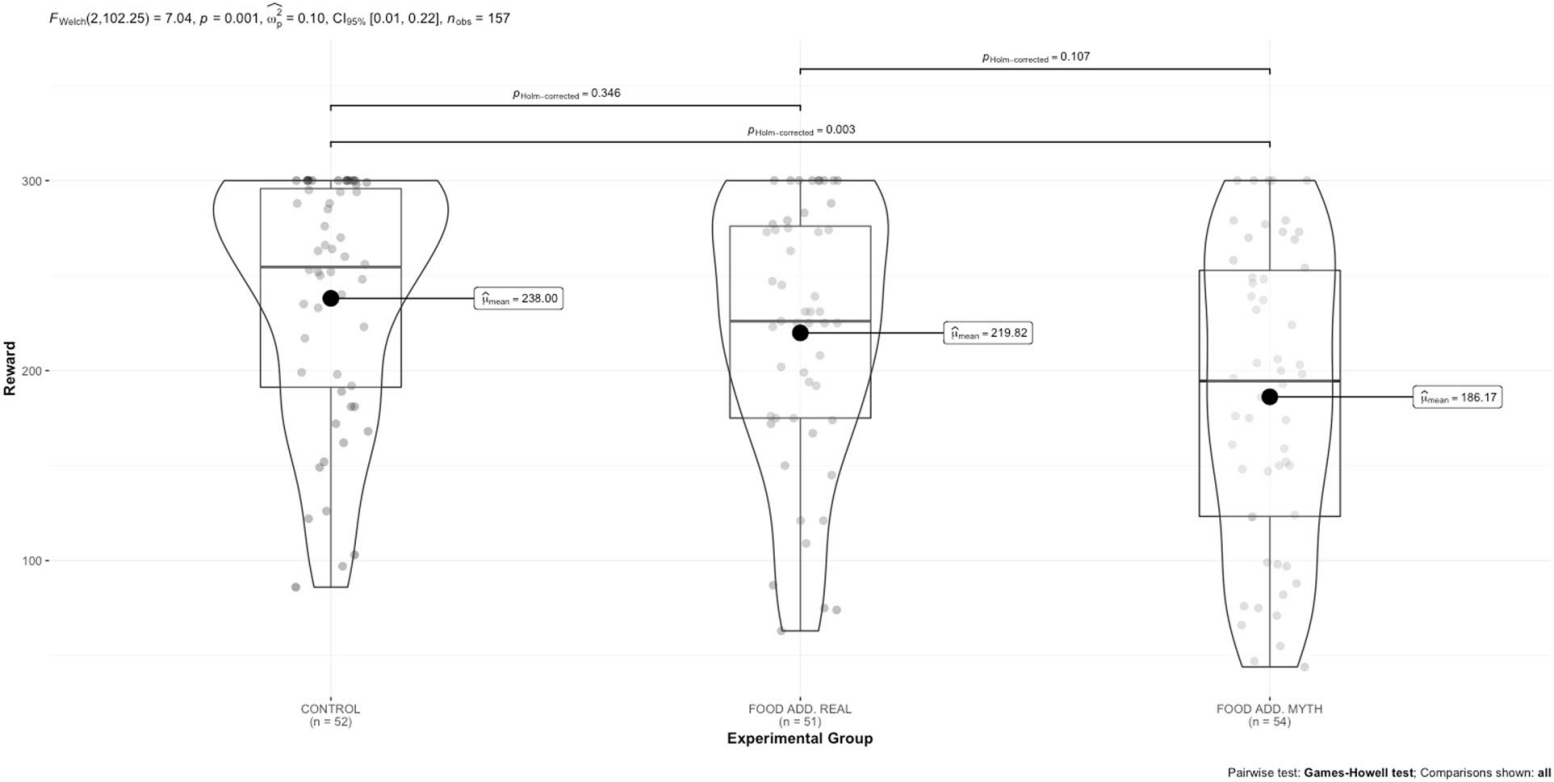
Differences in reward based on experimental group, in experiment 2. Points are individual data points. Boxplot represents the median and interquartile range. Violin plot represents the distribution of the data points.

When removing individuals who failed the manipulation check, the pattern of results did not change, see Fig. 2. Inclusion of a second between-subjects factor of sampling strategy (prolific vs local recruitment) in an exploratory analysis demonstrated no main effect or interaction by sampling (see online supplementary materials).

We collapsed the Food addiction Myth and Real groups and compared these to the control group to test the effect of learner weight status only on reward (Hypothesis 1). There was a significant difference between normal-weight learner (mean = 238.00, SD = 63.39) and overweight/obesity learner groups (mean = 202.51, SD = 75.18; *t*(118.57) = 3.10, *p* = 0.002 *g* = 0.49 [95% CI: 0.16 to 0.83], in that the learners with overweight/obesity were rewarded less than learners of normal weight.

#### Punishment

There was no significant effect of experimental condition on punishment (F(2107.40) = 0.66, *p* = .520, np2 = 0.00 [95% CI: ~0.00 to ~0.00]. Removal of individuals who failed the manipulation check did not substantially influence results (F(2101.31 = 0.92, *p* = 0.402, Np2 = 0.00 [95% CI: ~0.00 to ~0.00]. The average punishment in the control group was 69.96 (SD = 57.50); Food addiction myth group was 80.37 (SD = 74.25); and Food addiction real group was 60.98 (SD = 71.85).

When collapsing the groups to learners with normal weight (mean = 69.96, SD = 57.51) vs learners with overweight/obesity (mean = 70.95, SD = 73.39) there was no significant difference between the groups (*t*(126.19) = 0.09, *p* = 0.926, *g* = −0.01 [−0.35 to 0.32]).

#### Decisions to allow learners to progress

In the control group participants recommended the learners pass *N* = 42/52 (80.8%) times; in the Food Addiction Myth group participants recommended pass *N* = 38/54 (70.3%) times; in the Food Addiction Real group participants recommended pass *N* = 37/51 (72.5%) times. The Pearson’s chi-squared test was not significant (X^2^(2) = 1.66, *p* = 0.435), demonstrating no differences in the decisions to progress between the groups.

## Discussion

In two experiments we examined whether participants would financially reward or punish individuals based on perceived weight status, using a novel financial discrimination task. Across both studies there was some evidence of weight-bias, specifically in Experiment 1 individuals with overweight/obesity were punished to a greater extent than normal-weight individuals, and in Experiment 2 individuals with overweight/obesity were rewarded less than normal-weight individuals. Whilst our observed effect sizes were small-to-moderate, these findings were relatively robust against participants’ self-reported weight status (see online supplementary materials) and across sensitivity analyses.

Our findings support observational research providing evidence for weight-bias [10, 13]. However, these are the first experiments to our knowledge that have tested direct financial discrimination. Whilst these findings are based on small financial decisions in single sessions, it suggests that people readily discriminate against individuals with overweight/obesity, supporting previously established experimental evidence in hypothetical individuals for hiring/firing decisions [19, 20]. If these findings translated to decisions in which larger financial decisions were made (e.g. promotion or salary negotiations), then they may support the robust causal associations between weight status and income [17]. It is unclear why we observed greater punishment behaviours in Experiment 1 but lower reward behaviours in Experiment 2 for learners with overweight/obesity. It is possible this may be due to our sampling strategy in each study, and unknown participant ‘awareness’ of our aims. An alternative explanation for some of the lack of significant effects of learner weight status in Experiment 2 may be reduced statistical power due to the adoption of a between-subjects design. For example, there was a similar-sized and directional reduction in the percentage of participants permitting learners to progress associated with the learner having overweight/obesity in Experiment 1 (10.5%) and 2 (9.4%).

We also demonstrated no evidence that belief in food addiction as real versus a myth phenomenon influenced participant’s weight-bias to individuals whom they believed to have overweight/obesity. This is in contrast to previous studies which suggest priming beliefs about food addiction increases subsequent stigma [26].

There are several strengths to our research. Our novel platform allowed us to test direct discriminatory behaviour without informing participants that decisions were in fact hypothetical; thus overcoming any mental health/ethical considerations of measuring discrimination towards real people, whilst also reducing any issues with participant’s retrospective recall over long time periods [32].

This study also has several limitations. First, we tested a very specific discriminatory behaviour (financial reward/punishment) and therefore it is unknown whether these findings would replicate to other types of weight-bias (e.g. aggressive behaviour). Second, we were unable to test the interaction between learner weight status and food-addiction information in Experiment 2, as we did not use a fully balanced design. This decision was taken a-priori (as per our preregistration) to ensure we were adequately powered to detect our primary hypothesis with the resources available to us. Finally, we cannot rule out demand characteristics influencing our findings or a lack of belief in the cover story [33], because we did not specifically ask participants what they thought the study was about. Nevertheless, we did attempt to conceal the aims in Experiment 1 and the use of a between subject’s design in Experiment 2 reduces the likelihood of aim guessing.

This study presents several interesting avenues for future research. First, the salience of the weight-related information might be increased to examine if the magnitude of weight-bias is associated with the salience of the recipient’s weight (e.g. by using images, rather than biographies). Future studies might also attempt to directly assess participants’ beliefs about the nature of the experiment, e.g. using a funnelled debrief [34], or more explicitly measuring awareness of the research hypotheses (e.g. The Perceived Awareness of research Hypothesis Scale [35]). However, for Experiment 1 at least it is entirely possible that even if participants guessed the aim, they were still willing to demonstrate discrimination/anti-fat biases [36].

To conclude, using a novel paradigm we demonstrated evidence of weight bias in the form of financial discrimination, which was not influenced by information about food addiction. Effects were relatively small and varied across rewards and punishment. These findings support a growing body of evidence for the differential treatment of individuals of overweight/obesity, which might contribute to financial inequalities based on body weight.

## Supplementary information

Supplemental Material

## Data Availability

The datasets generated during and/or analysed during the current study are available in the OSF repository, [https://osf.io/pqzgs].
