# Supplementary material for: Weight-based discrimination in financial reward and punishment decision making: causal evidence using a novel experimental paradigm
Source: Int J Obes (Lond). 2022 Mar 25;46(7):1288–94. doi: 10.1038/s41366-022-01109-z (PMC9239905; doi:10.1038/s41366-022-01109-z)
Supplement: Supplementary file 1 — Supplemental Material [file 41366_2022_1109_MOESM1_ESM.docx]

Online Supplementary Materials:

Vignettes from Experiment 2:

FOOD ADDICTION MYTH

Many people find it very difficult to resist eating when they see or smell food. It is believed, by many, that certain foods are addictive, in much the same way as are drugs and alcohol. In fact, the existence of food addiction is widely disputed among the scientific community and a growing body of evidence indicates that foods – such as crisps – should not be put into the same category as drugs and alcohol. Although it is often argued that the “addictive” properties of certain foods are caused by high levels of salt, sugar and fat, experts say that there is very little evidence to support this theory. Many of the research studies have been conducted on laboratory rats and are clearly very different from the complex scenarios that are faced by the human eater.

There is no doubt that ingredients like salt and fat do make foods, such as crisps, taste good; however the idea that they drive us to continue eating is not correct. Research shows that “addictive” foods, such as crunchy salty crisps, do not affect the brain in the same way as drugs and tobacco. In fact, the only similarity is that both food and drugs stimulate the release of dopamine. This is not surprising because food is essential for our survival and drugs act by high-jacking the brain’s natural reward system. Prolonged drug use causes dramatic change in the brain; these sorts of changes are not seen in response to food and eating. These findings suggest that food addiction is merely a myth and it is wrong to blame the food industry for the rising levels of obesity

FOOD ADDICTION REAL

Many people find it very difficult to resist eating when they see or smell food. It is believed, by many, that certain foods are addictive, in much the same way as are drugs and alcohol. In fact, the existence of food addiction has been proven by the scientific community and a growing body of evidence puts foods – such as crisps – in the same category as drugs and alcohol. Experts now know that the addictive properties of certain foods are caused by high levels of salt, sugar, and fat, which are added by manufacturers. It is these ingredients that make the food taste so good and make it impossible for us to stop eating it even when we are full. This is particularly problematic because our bodies have not evolved to handle this over-stimulating concoction.

In times gone by, salt was a simple garnish and fat was a nutrient that had to be hunted or foraged. Nowadays we are bombarded with these ingredients and the effects on our bodies and brains are dramatic. Research shows that addictive foods, such as crunchy salty crisps, can trigger the brain in the same way as drugs and tobacco. The right combination of tastes stimulates the release of dopamine in the brain, which is known to be highly pleasurable. The release of dopamine motivates the eater to look for even more food. Over time, the brain’s reward system becomes even more sensitive to these effects and so the foods become increasingly harder to resist. These findings suggest that food addiction is a common occurrence and the way to help people eat less is to try and change the foods that are available to consumers.

CLIMATE CHANGE

Climate is sometimes mistaken for weather. But climate is different from weather because it is measured over a long period of time, whereas weather can change from day to day, or from year to year. The climate of an area includes seasonal temperature and rainfall averages, and wind patterns. Different places have different climates. A desert, for example, is referred to as an arid climate because little waterfalls, as rain or snow, during the year. Other types of climates include tropical climates, which are hot and humid, and temperate climates, which have warm summers and cooler winters.

The cause of current climate change is largely human activity, like burning fossil fuels, like natural gas, oil, and coal. Burning these materials releases what are called greenhouse gases into Earth’s atmosphere. There, these gases trap heat from the sun’s rays inside the atmosphere causing Earth’s average temperature to rise. This rise in the planet's temperature is called global warming. The warming of the planet impacts local and regional climates. Throughout Earth's history, climate has continually changed. When occurring naturally, this is a slow process that has taken place over hundreds and thousands of years. The human influenced climate change that is happening now is occurring at a much faster rate.

COGNITIVE TASK EXAMPLES


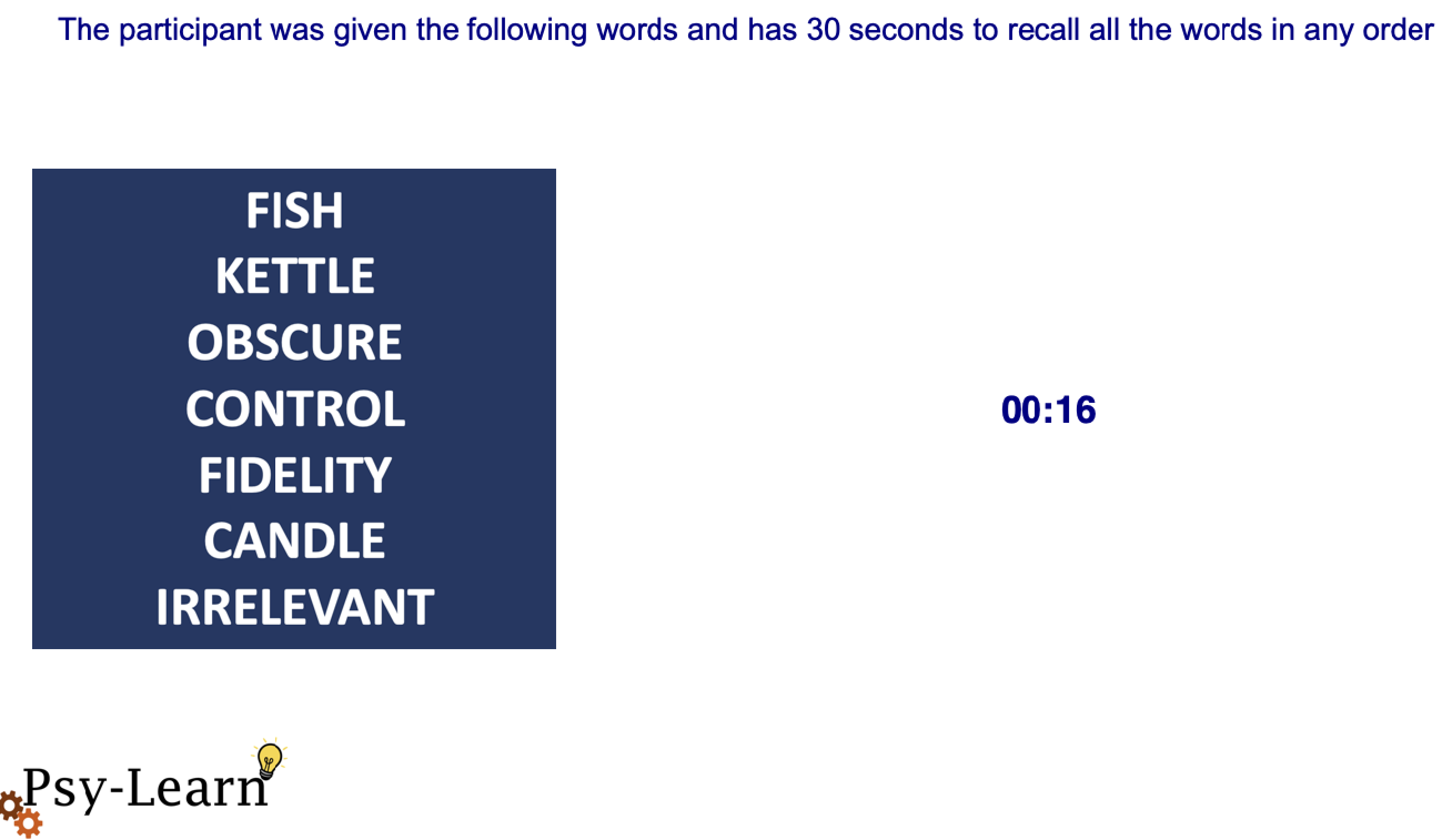


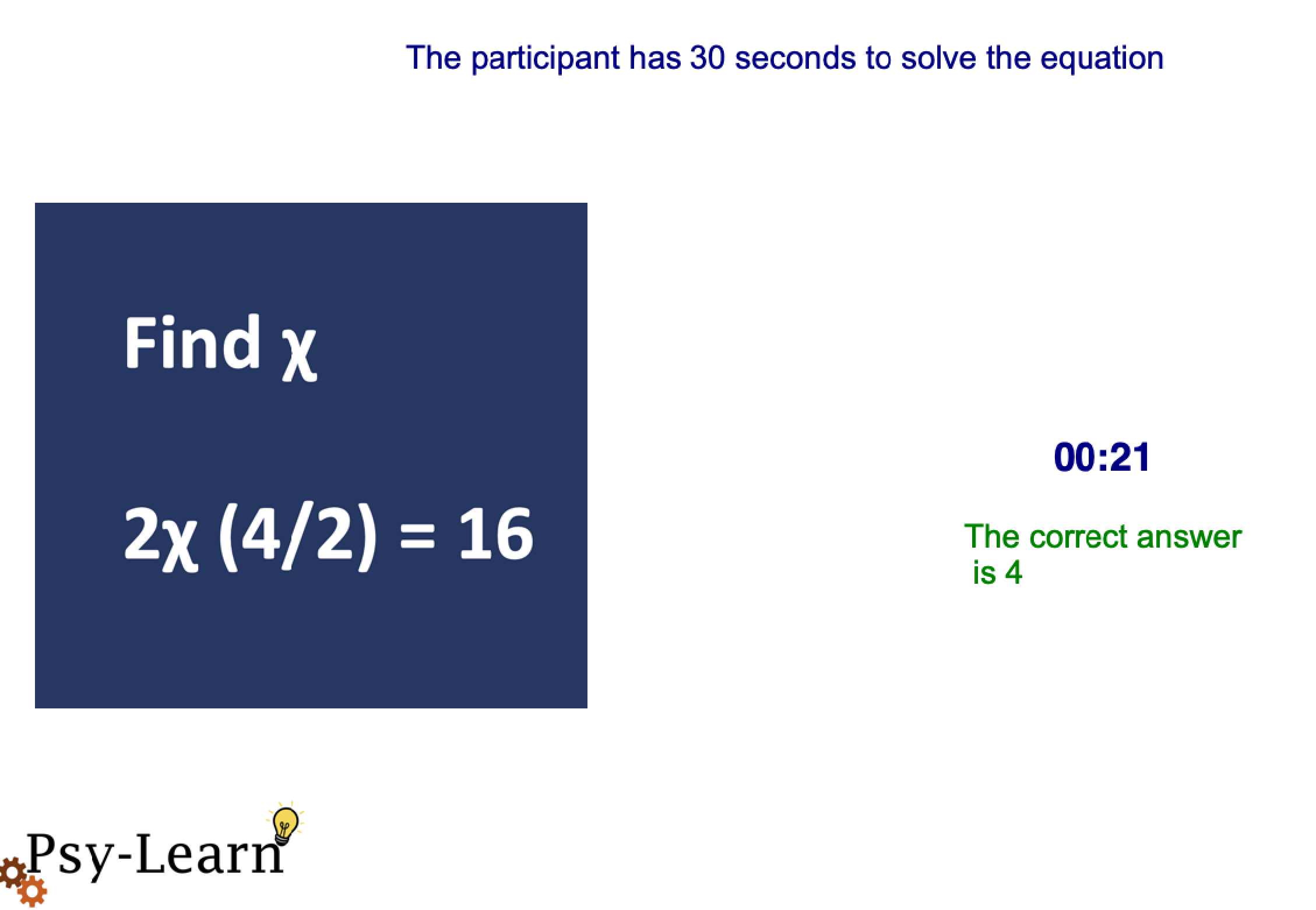


Trial flow:


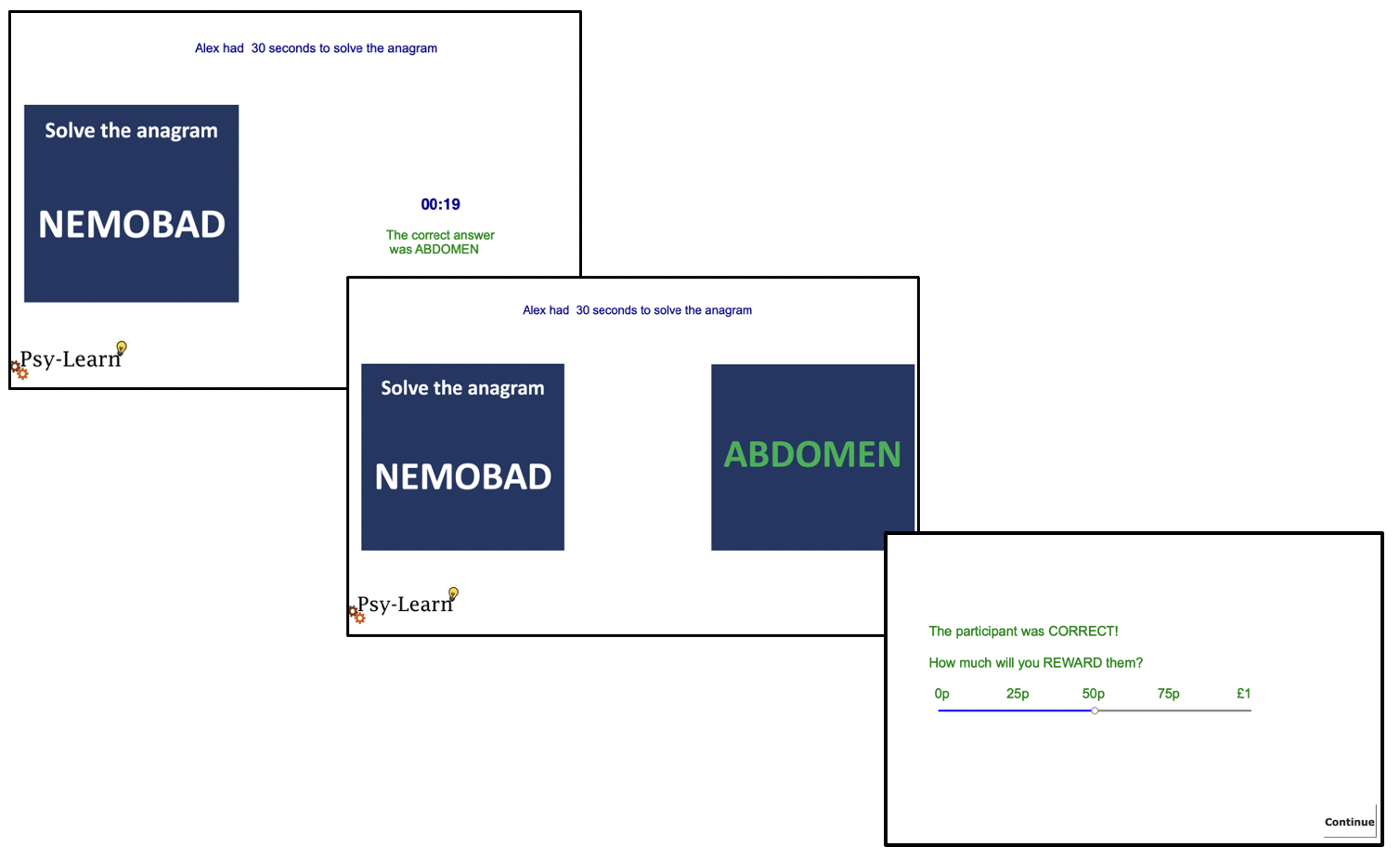


Boxplot for outliers in reward / punishment scores in experiment 1.


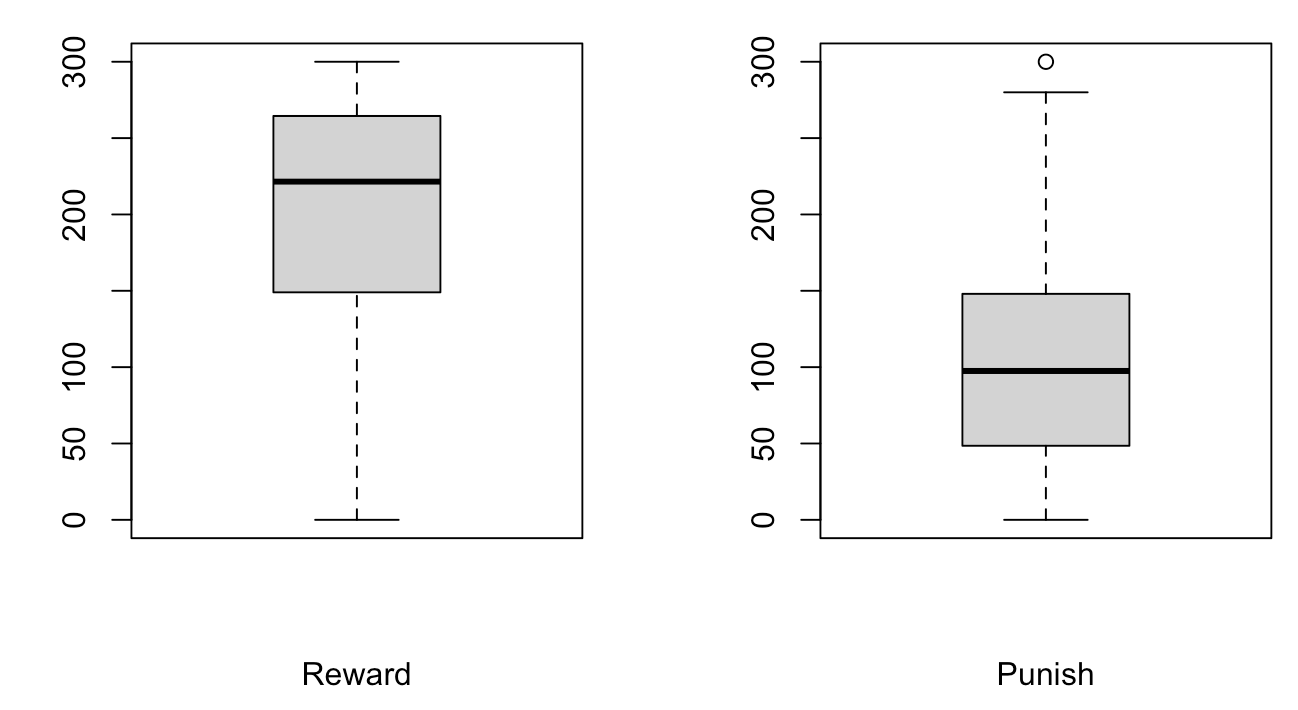


Boxplot for outliers in reward / punishment scores in experiment 2.


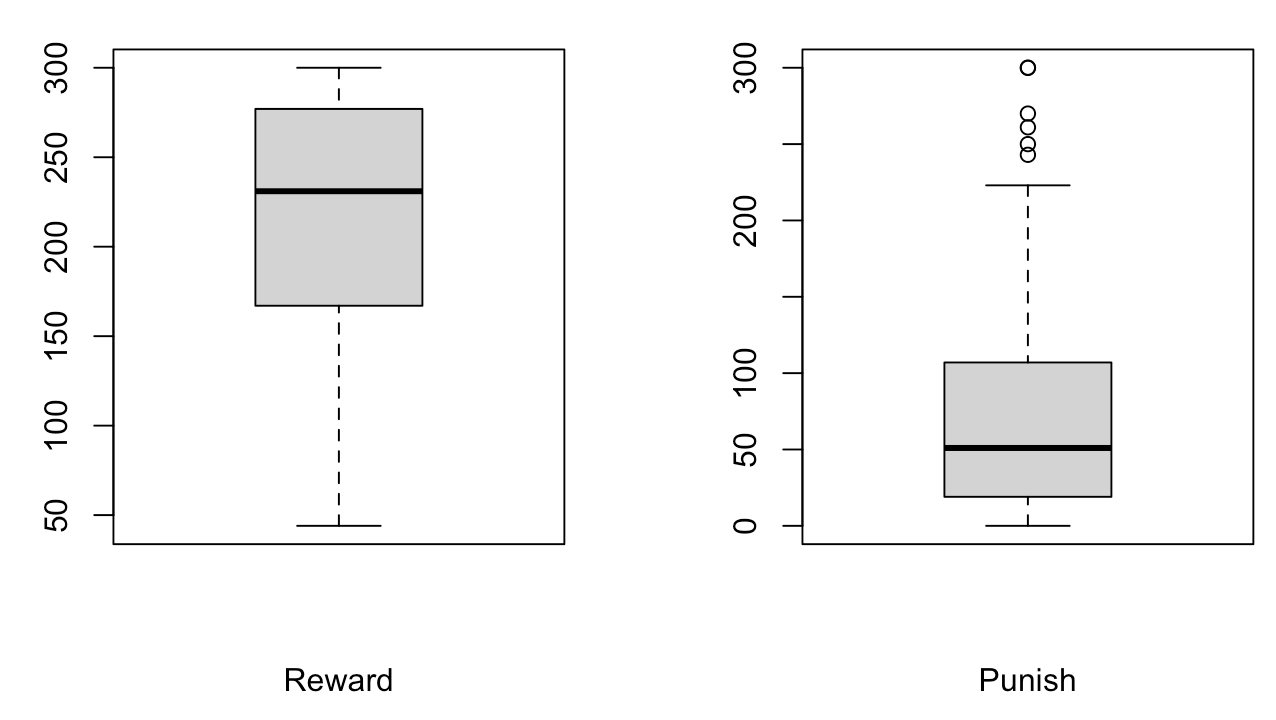


Experiment 1:

Repeated Measures ANOVA to model interactions

A repeated measures ANOVA was conducted with two factors (Behaviour: Reward vs Punishment, and Weight-Status: Normal Weight vs Overweight / Obese), on financial decisions (DV of 0 – 300 pence). There was a main effect of behaviour (F(1,105) = 150.91, p < .001) suggesting the magnitudes of rewards were greater than punishments, and a main effect of Weight-Status. There was no interaction (F(1,150) = 1.00, p = .319).

Effects in healthy weight participants only

As most participants self-reported normal weight status, we reanalysed the data from individuals who passed the manipulation check and self-reported normal weight. There was no significant difference in the reward between normal and overweight / obese learners (t(85) = 0.515, p = .608, g = -0.04 [95% CI: -.19 to 0.11]). For punishment, the significant difference between normal weight and overweight / obese learners remained (t(85) = 2.22, p = .029, g = -0.17 [95% CI: -0.02 to -0.31])

Reliability of Reward and Punishment (experiment 1)

We computed McDonald’s Omega as a measure of internal consistency of the reward and punishment on individual trials. Across all trial types, Omega was high suggesting a consistency in responses from participants (reward normal weight ω = .84; reward overweight / obese ω = .89; punishment normal weight ω = .81; punishment overweight / obese ω = .83)

Experiment 2:

Mixed Measures ANOVA to model interactions

A mixed ANOVA with a repeated measures variable of behaviour (Reward vs Punishment) and a between subjects’ factor of experimental group (Control + Normal weight vs Food Addiction Real + Obese vs Food Addiction Myth + Obese) was performed on financial decisions (0 – 300p). There was a main effect of reward vs punishment (F(1, 154) = = 309.65, p < .001), in that rewards were significantly larger than punishments. There was also a significant interaction (F(2,154) = 5.70 , p = .004). This suggests the effect of rewards are bigger than the effect of punishments within the specific experimental groups, outlined in the main findings.

Effects in healthy weight participants only

As most participants self-reported normal weight status, we reanalysed the data from individuals who passed the manipulation check and self-reported normal weight (N = 111). The effect of experimental group on reward was significant (F(2, 67.52) = 3.73, p = .029, Np2 = .07 [95% CI: .00 to .20]. However, none of the corrected comparisons were significant (ps > .111). The effect on punishment remained non-significant (F(2, 68.48) = 1.27, p = .287, np2 = .001 [95% CI: .00 to .07]).

Reliability of Reward and Punishment (experiment 2)

As in experiment 1 the internal consistency of both reward (ω = .86) and punishment trials (ω = .90) was high.

Inclusion of sampling strategy as a second between-subjects factor (experiment 2)

A 3 (experimental group) x 2 (sampling strategy: prolific vs local recruitment) ANOVA was conducted on reward. There was a main effect of group (F(2, 160) = 7.83, p < .001). However, there was no main effect of sampling strategy (F(1,160) = 2.27, p = .134) or sampling strategy * experimental group (F(2, 160) = 0.69, p = .504).
